# Seven remarkable new fossil species of parasitoid wasps (Hymenoptera, Ichneumonidae) from the Eocene Messel Pit

**DOI:** 10.1371/journal.pone.0197477

**Published:** 2018-06-06

**Authors:** Tamara Spasojevic, Sonja Wedmann, Seraina Klopfstein

**Affiliations:** 1 Wirbellose Tiere, Naturhistorisches Museum Bern, Bern, Switzerland; 2 Institute of Ecology and Evolution, University of Bern, Bern, Switzerland; 3 Forschungsstation Grube Messel, Senckenberg Forschungsinstitut und Naturmuseum, Messel, Germany; Royal Belgian Institute of Natural Sciences, BELGIUM

## Abstract

Parasitoid wasps of the family Ichneumonidae are one of the most diverse and species-rich groups of organisms with a worldwide distribution. We here describe seven new ichneumonid fossil species and two new genera from a remarkable insect fossil site, the Eocene Messel Pit in Germany (~47Ma). The unique fossil preservation allows us to place five out of the seven new species unequivocally in extant subfamilies and genera. For the first time, lobed claws which are a clear synapomorphy for the subfamily Pimplinae, are observed in a fossil, making the newly described *Scambus fossilobus* sp. nov. the oldest unequivocal representative of the group. We also describe a fossil of Labeninae (*Trigonator macrocheirus* gen. et sp. nov.), an ichneumonid subfamily that was until now believed to be an exclusively Gondwanan element. Furthermore, the newly described *Rhyssella vera* sp. nov., *Xanthopimpla messelensis* sp. nov., and *X*. *praeclara* sp. nov. provide evidence that these extant genera date back as far as the Early/Middle Eocene. In contrast to the clear placement of most of the newly described species, we were unable to place *Polyhelictes bipolarus* gen. et sp. nov. and *Mesornatus markovici* gen. et sp. nov. in an ichneumonid subfamily, mostly due to the high levels of homoplasy found in this group. These findings on the one hand demonstrate the need for a more rigorous approach in the taxonomic placement of fossil ichneumonids, and on the other hand provide more precise minimum ages for several ichneumonid genera and subfamilies.

## Introduction

The parasitoid wasp family Ichneumonidae is one of the largest groups of organisms, with more than 24,000 species described up to date [1] and at least 60,000 estimated [2,3]. The members of this family show remarkable diversity in morphology, behaviour, and host choice, as well as parasitoid life style. Despite the high species richness and straightforward family identification due to the highly conserved wing venation pattern, the fossil record of Ichneumonidae is severely understudied. Around 240 amber and impression fossil species of ichneumonids have been described, but many of them require revision [1,4] and hundreds more await description in palaeontological collections around the world [5–7].

The oldest fossils of Ichneumonidae date back to the Early Cretaceous. These include representatives of the extinct subfamilies Palaeoichneumoninae Kopylov and Tanychorinae Rasnitsyn (the placement of the latter within the family is controversial) [5,8–10]. These two subfamilies are replaced in the fossil record of the Late Cretaceous by the recently described Novichneumoninae Li et al. [11] and by the morphologically rather heterogeneous subfamily Labenopimplinae Kopylov, which was suggested as a potential ancestor of the extant subfamily Labeninae Ashmead or, less likely, Pimplinae Wesmael [12]. The only known Cretaceous fossil that is currently placed in an extant subfamily, *Albertocryptus dossenus* McKellar et al. [13] from Canadian amber, is considered to be a labenine. This fossil is clearly an ichneumonid, but its placement within the subfamily Labeninae is questionable according to the authors of the original description [13].

In contrast, the Paleogene ichneumonid fauna is dominated by representatives of extant subfamilies: out of 45 recent subfamilies, 18 are reported from this period, with Pimplinae, Cryptinae Kirby and Ichneumoninae Latreille being the most frequent. However, the taxonomic placement of many taxa is uncertain and/or in need of revision [6]. This difficulty in sound taxonomic placement of ichneumonid fossils mostly results from the lack of interpretable synapomorphies, which is caused both by the fossilisation process and high levels of morphological homoplasy within ichneumonids [7,14,15].

The Messel Pit Fossil Site (“Grube Messel”) is a UNESCO World heritage site located about 10km northeast of the city of Darmstadt (State Hesse, Germany). It formed during the Eocene in a maar lake and is renowned for the extremely good preservation of its fossils. For example, some vertebrates show exceptionally preserved soft tissues like fur or feathers [16–19]. In insects from Messel, the preservation even of tiny morphological structures can be extremely good, with many details visible [17,20–22]. The preservation of structural colours is especially impressive, as it documents the detailed conservation of tissue nanostructures in insects [23,24]. Elaborate patterns of non-structural colours are also often conserved in insects from Messel, not in their original colour, but as different shades of light brown to black. Although the insects from Messel are compression fossils, their preservation rivals the preservation in amber.

Overviews of the fossil flora and fauna from Messel are given by Schaal & Ziegler [18], Koenigswald & Storch [17] and Gruber & Micklich [25]. The vertebrate fossils comprise mainly fishes [26]. Other vertebrates are much rarer, but they provide important insights into the former terrestrial ecosystem, like reptiles [27,28], birds [29,30], and mammals [16,31]. Invertebrates, especially insects, document a very high biodiversity [20,21,32,33], as do the plants [34].

In this paper we are able to place five out of seven newly described ichneumonid fossil species in extant subfamilies and even genera with high certainty. For the first time, we report (a) fossilized claws showing a basal lobe, a clear synapomorphy of the subfamily Pimplinae, (b) a species of the subfamily Labeninae from Eurasia, and (c) the oldest unequivocal fossil representative of the subfamily Rhyssinae. These findings provide sound minimum age estimates for the subfamilies Pimplinae, Labeninae and Rhyssinae and prove that several recent genera date back as far as the Early Eocene, for instance the enigmatic genus *Xanthopimpla* which to date has only been recorded from the latest Eocene. They thus move ichneumonid palaeontology forward considerably and provide a sound basis for molecular dating of the phylogenetic tree of these parasitoid wasps in the future.

## Materials and methods

### Geological setting

The Messel Pit Fossil Site lies on the eastern side of the Rhine rift valley within the Sprendlingen Horst, an uplifted Palaeozoic basement block framed by Cenozoic depressions. Volcanic activity during the transition from the Early to Middle Eocene around 49 to 47 Ma [35] led to the formation of several isolated volcanic and tectonic basins, one of them Messel [36–38]. Scientific drilling has proven that the former Lake Messel was a maar lake which formed due to phreatomagmatic activity [36,39]. The uppermost 94 m of the drilling core are finely laminated bituminous claystone (“oil shale”) sediments which form the main part of the so-called “Middle Messel Formation” [36]. All fossils investigated in this study originate from this Formation. The currently accessible strata of this formation can be subdivided with the local marker horizons M, alpha, beta, and gamma (from top to bottom).

Revised radiometric dating of a basalt fragment suggests an age for the eruption of slightly more than 48 million years ago (between 48.27±0.22 and 48.11±0.22 Ma) and an according age of the collected fossils of a bit more than 47 Ma [35,40].

The maar crater in which the lake formed had a depth of initially about 300 to 400m and an original diameter of about 1.5 km, possibly even of more than 2 km [41]. The lake that formed in the maar crater was initially holomictic and later became permanently meromictic. The finely laminated oil shale represents deep water sedimentation of the meromictic lake [42,43]. The clay of the oil shale consists mostly of smectites, while the organic part is mostly made up of cell walls of the green algae *Tetraedron* [42]. The sedimentation rate of the oil shale of the former lake was very low, with estimated rates between 0.1 and 0.2 mm/year [42]. Schulz et al. [39] and Lenz [44] calculated an average sedimentation rate of 0.14mm/year for the oil shale of the Middle Messel Formation. The deposited oil shale documents that former Lake Messel existed for about 1 million years [39,42].

### Specimens and morphological examination

All specimens described in this study are publicly deposited and accessible at the Senckenberg Research Station Messel, Senckenberg Research Institute Frankfurt, Germany (SF) under following accession numbers: SF MeI 8814, SF MeI 13431, SF MeI 15245, SF MeI 16069, SF MeI 16962, SF MeI 16988, SF MeI 17300, SF MeI 17304. The fossils are stored permanently in glycerol to prevent damage by desiccation. All necessary permits were obtained for the described study, which complied with all relevant regulations. In all years in which the Senckenberg Research institute Frankfurt dug for fossils in the Messel Pit Fossil Site, it had permission to do so issued by the following authority: Archäologische und paläontologische Denkmalpflege, hessenArchäologie, Schloss Biebrich/Ostflügel, 65203 Wiesbaden, Germany.

All specimens were studied under a Leica MZ165 or a Leica MZ12.5 stereomicroscope and below glycerin. Photographs were taken either using a Leica MZ165 stereomicroscope with an attached Leica DFC 495 digital camera or a Leica MZ12.5 stereomicroscope with an attached Nikon D300 camera. Line drawings were prepared by overlaying the original image in Adobe Photoshop CS4. To describe general morphology, we used the terminology defined in Goulet & Huber [45], while the wing venation terminology was as defined in Spasojevic et al. [7]. All measurements were done directly on the images using ImageJ. Body length was measured from the apex of the head to the tip of the metasoma (excluding the ovipositor). The ovipositor length was measured as the length of the ovipositor sheaths and/or full length of ovipositor from the most anterior point of the base. Unless stated otherwise, measurements represent lengths of the respective structures. We used letters “H” and “P” to denote when a character is visible only in the holotype or only in the paratype, respectively. When two measurements are given e.g., 2Rs 0.7x/0.6x 2 + 3M, the first corresponds to the holotype and the second to the paratype. Additional abbreviations and explanations: T = tergite, S = sternite, areolet = forewing area surrounded by veins 2Rs, 2 + 3M, 4M, 3rs-m and, when areolet is pentagonal, by 3Rs.

The interpretation of many of the morphological characters was quite straightforward. Minor difficulties were present when interpreting, for example, the orientation of the metasoma, the number of bullae in the forewing vein 2m-cu, or the presence of carinae on the propodeum. In such cases, we always expressed that uncertainty in the descriptions and indicated it as dotted lines in the drawings. Although the preserved colouration itself is not original, we considered patterns of colouration as reflecting the state in live animals. Comparable colour patterns are also present in extant taxa.

We followed the subfamily classification of Ichneumonidae as given in Broad et al. [46] and for the lower taxonomic levels as referenced in Yu et al. [1], with a few exceptions that are clearly stated in the “Systematic placement” section. Additionally, we took extra care to avoid poorly supported taxonomic placements of fossils, making use of the nomenclature from open taxonomy [7,47] and describing new genera within Ichneumonidae *incertae subfamiliae* when there was not sufficient evidence for placing them in extant subfamilies.

### Nomenclatural acts

The electronic edition of this article conforms to the requirements of the amended International Code of Zoological Nomenclature, and hence the new names contained herein are available under that Code from the electronic edition of this article. This published work and the nomenclatural acts it contains have been registered in ZooBank, the online registration system for the ICZN. The ZooBank LSIDs (Life Science Identifiers) can be resolved and the associated information viewed through any standard web browser by appending the LSID to the prefix “http://zoobank.org/”. The LSID for this publication is: urn:lsid:zoobank.org:pub:3CE34A09-336D-42D6-A579-CAF41BDA0D4D. The electronic edition of this work was published in a journal with an ISSN, and has been archived and is available from the following digital repositories: PubMed Central, LOCKSS, ResearchGate.

### Systematic palaeontology

**Subfamily** Pimplinae Wesmael, 1844 [48]

**Tribe** Ephialtini Hellén, 1915 [49]

**Genus** Scambus Hartig, 1838 [50]

*Scambus fossilobus* sp. nov.

urn:lsid:zoobank.org:act:D90B0616-748A-4119-B024-725E98C90D27

(Figs 1 and 2)

**Fig 1:**
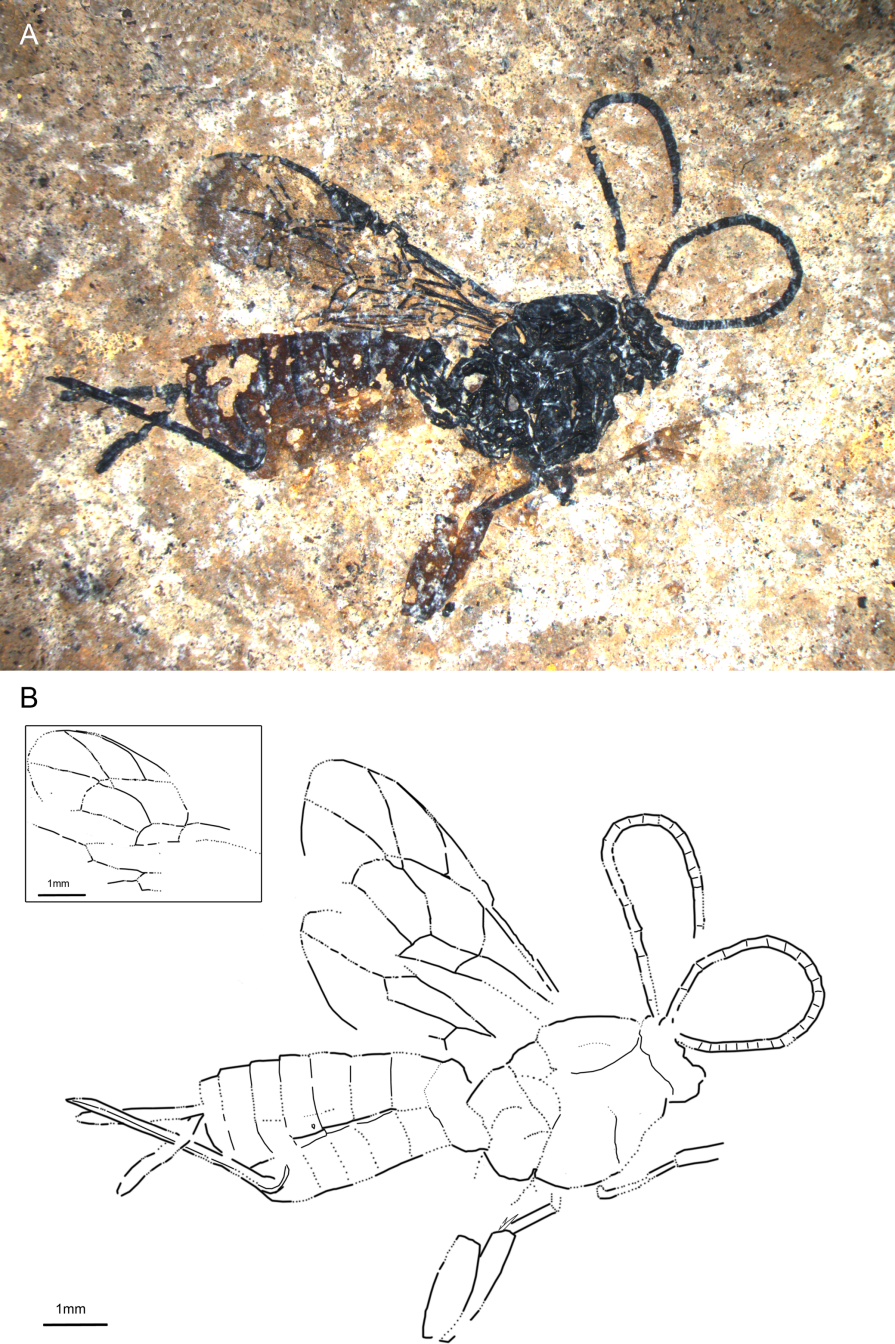
*Scambus fossilobus* sp. nov., holotype SF MeI 13431. (A) Photograph. (B) Drawing; left and right fore and hind wings were separated in the drawing; dashed lines indicate uncertain interpretations.

**Fig 2:**
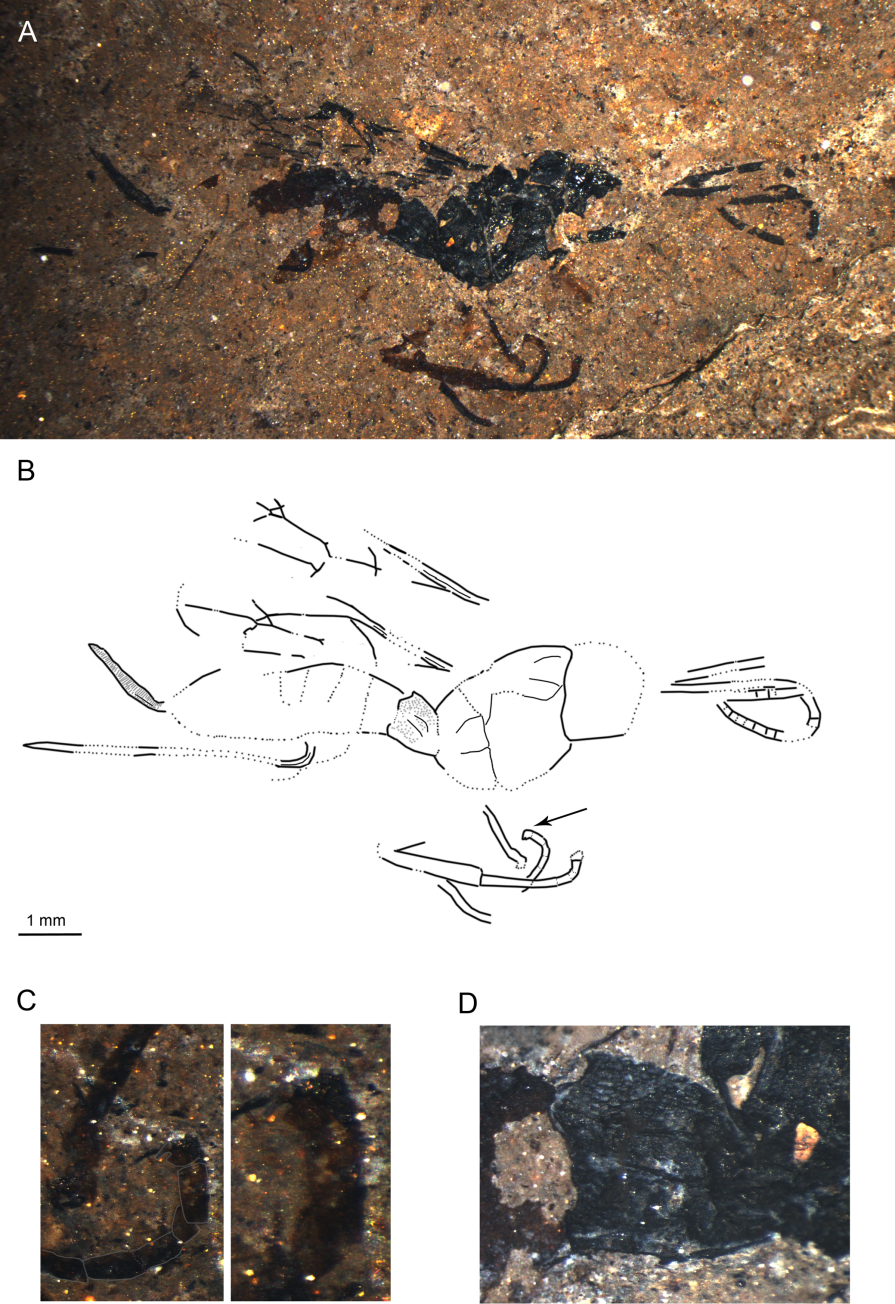
*Scambus fossilobus* sp. nov., paratype SF MeI 16962. (A) Photograph. (B) Drawing; left and right forewings were separated in the drawing; dashed lines indicate uncertain interpretations, grey dots indicate sculpture, and the arrow indicates the position of the lobed claw. (C) Outlined lobed claw on the middle leg of the fossil. (D) Tergite 1 of the metasoma with details of sculpture.

#### Etymology

From the Latin words for fossil “fossile” and lobe “lobus”, making reference to the first record of a basal lobe on a claw in an ichneumonid fossil.

#### Studied material

Holotype: SF MeI 13431, female. Dorsolateral aspect of body with antennae, forewings, incomplete hind wings, parts of fore legs, hind leg and ovipositor; hind leg preserved from femur to tarsal segment 1 with traces of coxa. Paratype: SF MeI 16962, female; dorsolateral aspect of body with incomplete antennae and forewings, traces of hind wings, parts of hind and mid legs including claws, and ovipositor; parts of head, mesosoma and metasoma missing.

#### Type locality and horizon

Messel Pit Fossil Site (Hesse, Germany), Middle Messel Formation. Details for holotype SF MeI 13431: grid square E 8/9, 3.5m to 4.5m above local stratigraphic marker level alpha. Details for paratype SF MeI 16962: grid square F 8/9, 2.5m to 2.85m above local stratigraphic marker level alpha.

#### Systematic placement

The specimens clearly belong to the subfamily Pimplinae based on the presence of lobes on the claws (see also “Remarks”), the broadly sessile T1, medium ovipositor length and forewing with quadrate areolet and two bullae in 2m-cu [46,51]. Within the subfamily Pimplinae, lobed claws occur in most of the representatives of the tribe Ephialtini and in the genera *Apechthis* Förster and *Itoplectis* Förster of the tribe Pimplini Wesmael. The fossil can be distinguished from all the Pimplini by the hind wing vein 1Cu being longer than cu-a and, additionally, by the straight tip of the ovipositor from *Apechthis*. Within Ephialtini, many genera can be excluded based on forewing and ovipositor lengths and on the ratio of 1Cu and cu-a in the hind wing [2]. The fossils particularly resemble the genus *Scambus* and the related genus *Endromopoda* Hellén, with a 1Cu/cu-a ratio in the hind wing of more than 1.5, the ovipositor shorter than half the length of the metasoma, and the strong punctuation of the mesosoma and T1 [52–54]. The oblique teeth visible on the lower valve of the ovipositor clearly place the fossil in the genus *Scambus*.

#### Diagnosis

At least middle legs with tarsal claws bearing basal lobe. Ovipositor around 0.4x as long as forewing, apex with oblique teeth. Areolet quadrate with uneven sides; hind wing vein 1Cu longer than 1cu-a. T1 short and stout, coarsely punctured in posterior half and with dorsal longitudinal carinae.

The fossil differs from *S*. *mandibularis* Spasojevic et al. [7] from the Eocene Green River formation by the shorter ovipositor, different areolet vein proportions and orange-brown hind coxae, while it has a slightly longer ovipositor and different areolet proportions than *S*. *parachuti* Spasojevic et al. [7]. *S*. *fossilobus* clearly differs from “*Scambus*” *fossilis* Khalaim in size (forewing length 3.8 cm in “*Scambus*” *fossilis*), the narrower cell 2R1 and higher 3Cu/2cu-a ratio [55]. Comparison of the fossil with all the extant *Scambus* species is difficult [6], but from most of them it can be distinguished by the combination of (1) ovipositor about as long as hind tibia, (2) entirely black mesosoma, (3) T2–T6 distinctly lighter than T1, which is concolorous with the mesosoma, (4) entirely dark pterostigma [52–54].

#### Description

Ground colour black. Legs orange-brown with tarsal segments darker on dorsal and lighter on ventral side (P). T1 black, rest of tergites orange-brown (T3–T7 appear to have dark band in apical one-fifth, but as result of overlap with preceding tergites (H)). Ovipositor and ovipositor sheaths black.

Head with antennae incomplete, with at least 24 flagellomeres (H); first flagellomere around 4.3x as long as wide (H), following flagellomeres elongate, later sub-quadrate; scape and pedicel not discernible.

Mesosoma with mesoscutum either smooth (H) or with small dense punctures (P) (sculpture difference likely preservation artefact, with punctured state probably being correct one). Notauli present. Pronotum about as long as deep (H). Epicnemical carina extending at least slightly below mid height of pronotum. Propodeum short, higher than long, with at least two longitudinal carinae that either correspond to pleural and lateral longitudinal carina or pleural and submetapleural carina; another longitudinal carina on top might be present which could correspond to medial or lateral longitudinal carina.

Forewing with areolet quadrate with uneven sides, 2Rs 0.7x/0.6x 2 + 3M and 0.8 rs-m, 2 + 3M 6.1x/4.9x 4M (vein in holotype not clearly visible), 3Rs at most as long as width of surrounding veins. 4Rs straight proximally, bending downwards at its distal half (H). Pterostigma around 3x as long as deep, 0.8 x 1R1 (H). 2Cu around 1.1x 1M&1Rs and r-rs (H). 1Rs + M (ramulus) as long as width of surrounding veins (P) or absent (in holotype not clear if absent or short). 3Cu 1.2x 2cu-a (H). 1m-cu at the junction of M + Cu and 1M. 2m-cu bowing outwards, probably with two bullae. Hind wing 1Cu/cu-a ratio 1.7x, 1Rs 1.5x rs-m (H).

Legs with hind femur around 2.7x as long as wide (H); hind tibia 5.2x as long as wide with two tibial spurs long and pointed (H); first tarsal segment 5.4x as long as broad, second segment around 2.8x as long as broad, third segment 1.8x as long as broad, fourth tarsal segment a bit shorter than wide, fifth 2x as long as broad (P). Tarsal segments of middle legs with claw with basal lobe (P).

Metasoma with T1 short, stout, strongly tapered in basal half,more or less parallel sided in apical half (P); dorsal longitudinal carinae present, extending over entire length; surface at least in posterior 2/3 strongly and coarsely punctured. T2–T5 subquadrate to transverse; spiracles clearly visible on T3, T5 and T6; clear crease between T2–T6, laterotergites rather narrow (H). Ovipositor stout, parallel sided with pointed apex and with oblique teeth on lower valve, 0.35/0.38x forewing length; ovipositor sheaths strongly wrinkled (H).

Measurements: forewing = 5.6/5.2 mm; body = 8.3/7.7 mm; hind tibia = 2/1.8 mm; metasoma = 5/4.6 mm; T1 = 1.2/0.9 mm; T2 = 1.4 mm (H); ovipositor = 3.7 mm (H); ovipositor sheaths = 2.1/1.8 mm.

#### Remarks

Lobed claws also occur in the tribe Poecilocryptini Townes and Townes of the subfamily Labeninae [2] and in the subfamilies Orthopelmatinae Schmiedeknecht and Collyriinae Cushman [46], all of which have a very distinct appearance. Poecilocryptini can be excluded based on them having only one bulla in 2m-cu, a pentagonal or open (3rs-m absent) areolet, and a longer and more slender T1 and ovipositor; Orthopelmatinae based on their small size, forewing with open areolet, deep pterostigma and very deep cell 2R1, and the hind wing without vein 2Cu; and Collyriinae based on their elongate propodeum and T1, and the ovipositor evenly tapered and bent with ventral teeth on entire length [2,46].

Tribe **Pimplini** Wesmael, 1844 [48]

Genus ***Xanthopimpla*** Saussure, 1890 [56]

***Xanthopimpla messelensis*** sp. nov.

urn:lsid:zoobank.org:act:1F7DC47E-FBE0-44D5-846F-FCE58272294B

(Fig 3)

**Fig 3:**
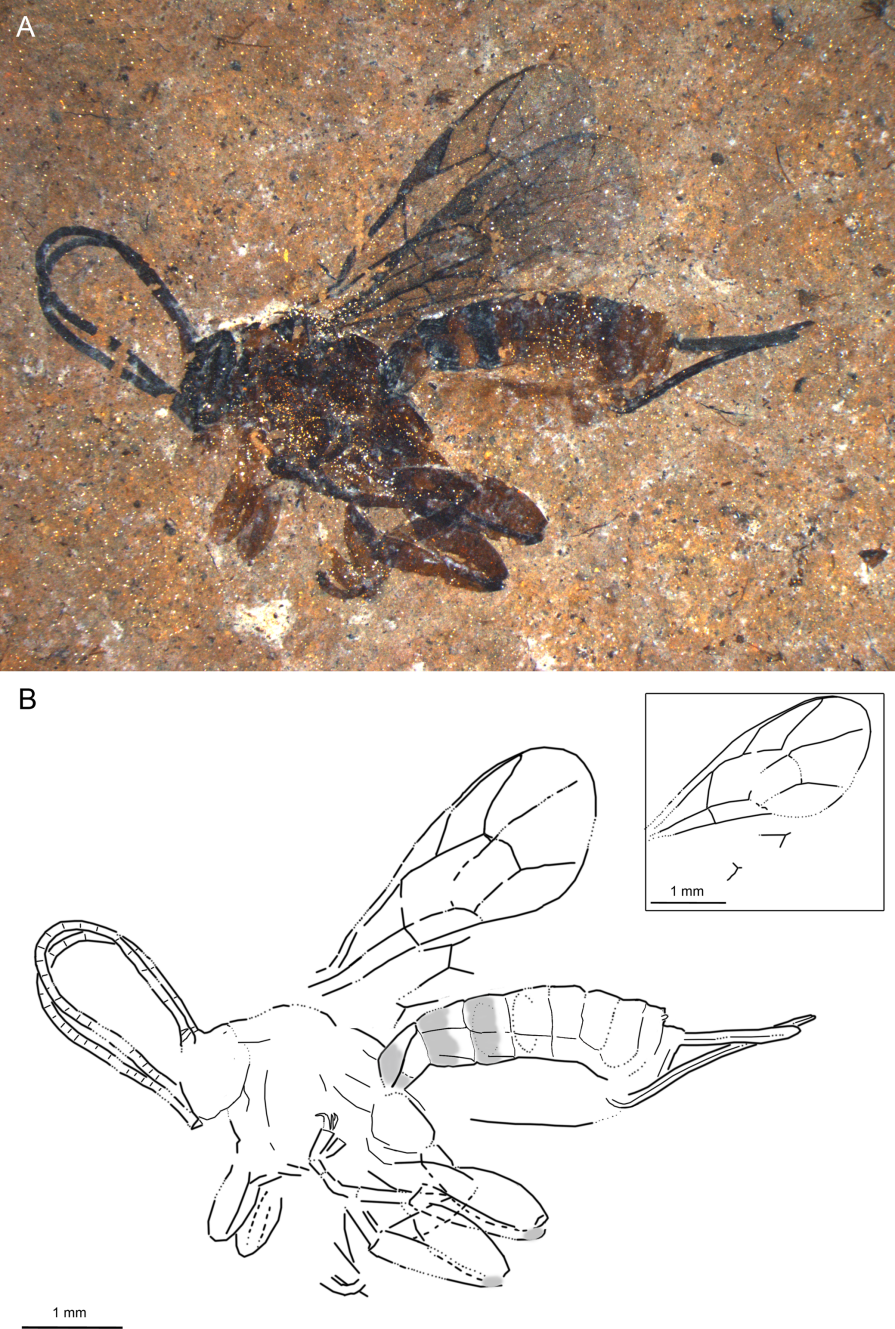
*Xanthopimpla messelensis* sp. nov., holotype SF MeI 16988. (A) Photograph. (B) Drawing; left and right fore and hind wings were separated in the drawing; dashed lines indicate uncertain interpretations, while grey areas indicate colour patterns.

#### Etymology

Named after the excavation locality, the Messel Pit.

#### Studied material

Holotype: SF MeI 16988, female. Lateral aspect of body with almost complete antennae, forewings, parts of hind wings, almost complete legs, and ovipositor.

#### Type locality and horizon

Messel Pit Fossil Site (Hesse, Germany), Middle Messel Formation. Details for holotype SF MeI 16988: grid square F 8/9, 2.05m to 2.5m above local stratigraphic marker level alpha.

#### Systematic placement

Based on the short and stout T1, two bullae in 2m-cu, the low ratio of 1Cu/cu-a in the hind wing and medium length ovipositor, this specimen can firmly be placed in the subfamily Pimplinae [46,51]. The fossil mostly resembles the Pimplini genera around *Theronia* Holmgren and *Xanthopimpla* in general appearance, especially with respect to the colour pattern, thickened legs and enlarged tarsal claws. The de-curved ovipositor, S-curved 2m-cu, very thick hind femur and conspicuous black base of the hind tibia suggest the genus *Xanthopimpla* [57,58]. If the mandible has been interpreted correctly, then the single visible tooth is further evidence for this placement (the mandible is twisted in *Xanthopimpla* so that only the upper tooth is visible when the mandible is closed).

#### Diagnosis

Ovipositor robust, weakly down-curved around middle; ovipositor sheaths around 0.36x forewing length. Areolet very narrow, triangular, slightly petiolate. Ground colour dark orange-brown; mesosoma with black markings on mesoscutum; T1–T3 with anterior half black, T6 with some black markings. T3–T4 with raised rhomboidal central area. Hind femura thickened. Hind wing 1Cu/cu-a ratio less than 0.5; 1Rs slightly longer than rs-m. 2m-cu curved in anterior half with two bullae; 1cu-a at junction of M + Cu and 1M.

*X*. *messelensis* sp. nov. differs from the fossil *X*. *biamosa* Khalaim and all recent *Xanthopimpla* species by the combination of (a) more narrow areolet, (b) black patterns present on T1–T3 and T6, (c) ovipositor de-curved on entire length and around 0.36x as long as forewing and (d) its small size [57–59].

#### Description

Ground colour dark orange-brown. Antennae black. Mesosoma with black markings on mesoscutum. Pterostigma and wing veins dark brown. Hind legs same as ground colour, but hind tibia with base, and possibly apex as well, darker; fore and mid legs slightly lighter. Anterior half of T1–T3 black; T4–T5 more or less uniformly coloured orange-brown, T6 with some darker pattern. Ovipositor and ovipositor sheaths dark brown to black.

Head short, with antennae almost complete, with at least 24 flagellomeres, most apical ones subquadrate; pedicel conspicuously smaller than scape, slightly wider than first flagellomere. One mandibula visible, seemingly with one tooth.

Mesosoma shortened, especially mesoscutum. Pronotum higher than long, with long carina curved around middle—most probably epomia. Propodeum with pleural carina, lateral and/or medial longitudinal carina.

Areolet narrow, triangular, slightly petiolate, 2Rs 1.2x 2 + 3M and 0.9x 3rs-m, 4M absent. 4Rs curved proximally. Pterostigma 3.7x as long as deep, 0.9x 1R1. 1m-cu&2Rs + M arched or slightly humped; 1Rs + M absent. 2Cu 1.2x 1M + 1Rs, 1x r-rs. 1cu-a at junction of M + Cu and 1M. 3Cu 0.9x 2cu-a. 2m-cu curved in upper half, probably with two bullae. Hind wing 1Cu/cu-a ratio 0.4, 1Rs 1.7x rs-m.

Hind coxa around 0.9x as long as high. Hind femur 2.8x as long as wide. Hind tibia 4.6x as long as wide. Hind claws enlarged, with elongated pulvillus.

Metasoma mostly in lateroventral aspect; T2–T4 appear to be in ventral aspect, based on dark colouration in proximal part, with some laterotergites visible. T1 short, 1.36x T2; lateral and dorsal longitudinal carina might be present; S1 if present around 0.3x T1. T2 subquadrate to transverse, remaining tergites transverse; T3 and T4 with raised rhomboidal central area. Ovipositor robust, weakly down-curved around middle; ovipositor sheaths wrinkled, 0.36x forewing length.

Measurements: forewing = 3.6 mm, width = 1.4 mm; body = 4.7 mm; metasoma = 3.2 mm; T1 = 0.7 mm; T2 = 0.5 mm; ovipositor = 2.2 mm; ovipositor sheaths = 1.3 mm.

***Xanthopimpla praeclara*** sp. nov.

urn:lsid:zoobank.org:act:B5AB5D62-573B-4618-8304-51F95DB8F67B

(Fig 4)

**Fig 4:**
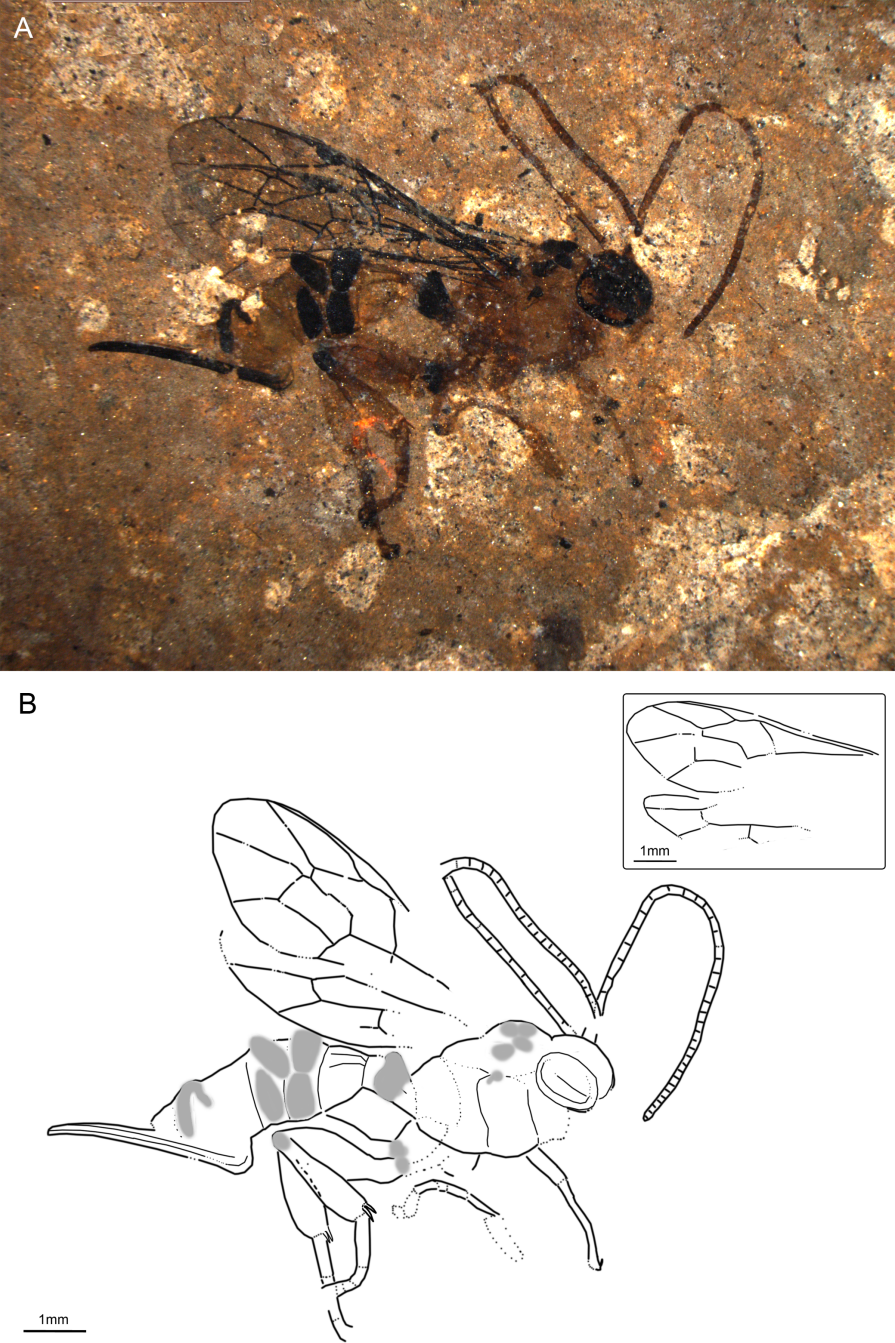
*Xanthopimpla praeclara* sp. nov., holotype SF MeI 17300. (A) Photograph. (B) Drawing; left and right fore and hind wings were separated in the drawing; dashed lines indicate uncertain interpretations, while grey areas indicate colour patterns.

#### Etymology

From Latin “praeclarus” which means “excellent”, “striking”, “distinguished”.

#### Studied material

Holotype: SF MeI 17300. Dorsolateral aspect of body with antennae, forewings, incomplete hind wings, hind legs, parts of fore and mid legs, ovipositor and ovipositor sheaths.

#### Type locality and horizon

Messel Pit Fossil Site (Hesse, Germany), Middle Messel Formation. Details for holotype SF MeI 17300: grid square F 8/9, 2.05m to 2.5m above local stratigraphic marker level alpha.

#### Systematic placement

The specimen can firmly be placed in the subfamily Pimplinae based on the short T1 with dorsal carinae, forewing with 2Rs longer than 2 + 3M and M + Cu at junction of 1M and 1cu-a, and medium long ovipositor [46,51]. The very distinctive colour pattern closely resembles a pattern seen in most extant *Xanthopimpla* species [57,58]. Together with the down-curved ovipositor and strongly thickened legs, this gives high support for placing the fossil in this genus.

#### Diagnosis

Ground colour light orange-brown. Mesoscutum with four black patches, plus one black patch in front of scutellum and one at base of forewing. Hind trochanter and base of hind tibia also black. T3 and T4 each with two lateral black patches; dorsal part of last tergites dark. Hind coxa wider than deep; hind femur around 2.5x as long as wide; hind tibia around 4x as long as wide, with short apical spurs. T1 short with dorsal longitudinal carinae. Ovipositor around 0.3x forewing length, slightly down-curved in posterior half. Areolet open pentagonal. 1Cu about as long as cu-a.

The fossil differs from the only known fossil species of *Xanthopimpla*, *X*. *biamosa* Khalaim, in the open areolet, paired colour marks on T4 and T6 (and maybe T7), straight 2m-cu, size (forewing 8mm long in *X*. *biamosa*), shorter ovipositor (0.8x metasoma length in *X*. *biamosa*), ratio 1Cu and cu-a in the hind wing, and the colouration of hind legs (no black markings in *X*. *biamosa*) [59]. Furthermore, *X*. *praeclara* sp. nov. can be distinguished from the newly described *X*. *messelensis* sp. nov. by the open areolet, straight 2m-cu, ratio of 1Cu and cu-a in the hind wing, ovipositor bent only from the posterior half, lighter ground colour and lack of black markings on the T2. It can be distinguished from the extant *Xanthopimpla* species by combination of the (a) open areolet and straight proximal section of 4Rs vein, (b) larger ovipositor sheaths/ hind tibia ratio and (c) colouration pattern of the metasoma [57,58].

#### Description

Ground colour light orange-brown with distinctive black markings. Head seems all black, inner part of complex eye lighter. Antennae orange-brown. Mesoscutum with four black patches, plus one black patch in front of scutellum and one at base of forewing (probably part of tegula). Hind trochanter and base of hind tibia also black. At least posterior half or entire T1 black except light orange-brown base and narrow band along apex; T3 and T4 each with two lateral black patches; dorsal part of last tergites dark. Ovipositor black, its sheaths orange-brown.

Head with antenna 1.2x times as long as forewing, with about 38 flagellomeres; apical flagellomeres transverse, medial ones about 1.2x as long as wide; short and abundant multiporous plate sensillae visible on many flagellomeres. Eye large, gena very narrow; inner margin of eyes probably more or less parallel. Two unidentate mandibles might be discernible.

Mesoscutum with notauli deeply impressed anterior but their length unclear. Sutures weak or invisible, except for side of mesoscutum. Subtegular ridge indicated. Epicnemical carina distinct below but unclear how far up. Propodeum around as long as high, possibly with closed petiolar area and additional carinae but interpretation uncertain.

Forewing vein 3rs-m absent (areolet open pentagonal), 2Rs 1.3x 2 + 3M. Pterostigma 3.5x as long as deep, 0.6x 1R1. 2Cu 0.8x 1M + 1Rs, 0.9x r-rs. 1m-cu&2Rs + M curved. 2m-cu more or less straight, probably with two bullae. 1cu-a at junction of M + Cu and 1M. 3Cu as long as 2cu-a. Hind wing 1Cu/cu-a ratio 1.1; 2Rs 2x rs-m.

Hind coxa short, inflated, wider than deep. Hind femur short, thick, 2.5x as long as wide. Hind tibia enlarged, 3.9x as long as wide, with short apical spurs—inner one slightly shorter than outer. Hind tarsal segments shortened; segment 5 0.9x as long as segment 1. Hind claws very big, without basal lobe; pulvillus between claws large.

Metasoma with T1 short, converging towards base, with median dorsal carinae which first converge and then go parallel until more than 0.5x of T1; T2 possibly with central swollen area; T3 and T4 with pair of black, heavily sclerotized and punctured patches, which are symmetrical in T3 but not T4 (probable artefact); sequence of tergites a bit unclear, probably some distortion. Ovipositor 0.29x forewing length, robust, slightly down-curved in posterior half; tip of ovipositor blunt, without notch,with at least four oblique teeth on ventral valve; base of ovipositor visible under T6/T7.

Measurements: antennae = 7 mm; forewing = 5.9 mm, width = 2 mm; body = 7.5 mm; metasoma = 4.5 mm; hind tibia = 1.7 mm; ovipositor = 3.4; ovipositor sheaths = 1.8 mm.

Subfamily **Rhyssinae** Morley, 1913 [60]

Genus ***Rhyssella*** Rohwer, 1920 [61]

***Rhyssella vera*** sp. nov.

urn:lsid:zoobank.org:act:6BE238D5-A45D-48A8-8E2F-DFE9010A5390

(Fig 5)

**Fig 5:**
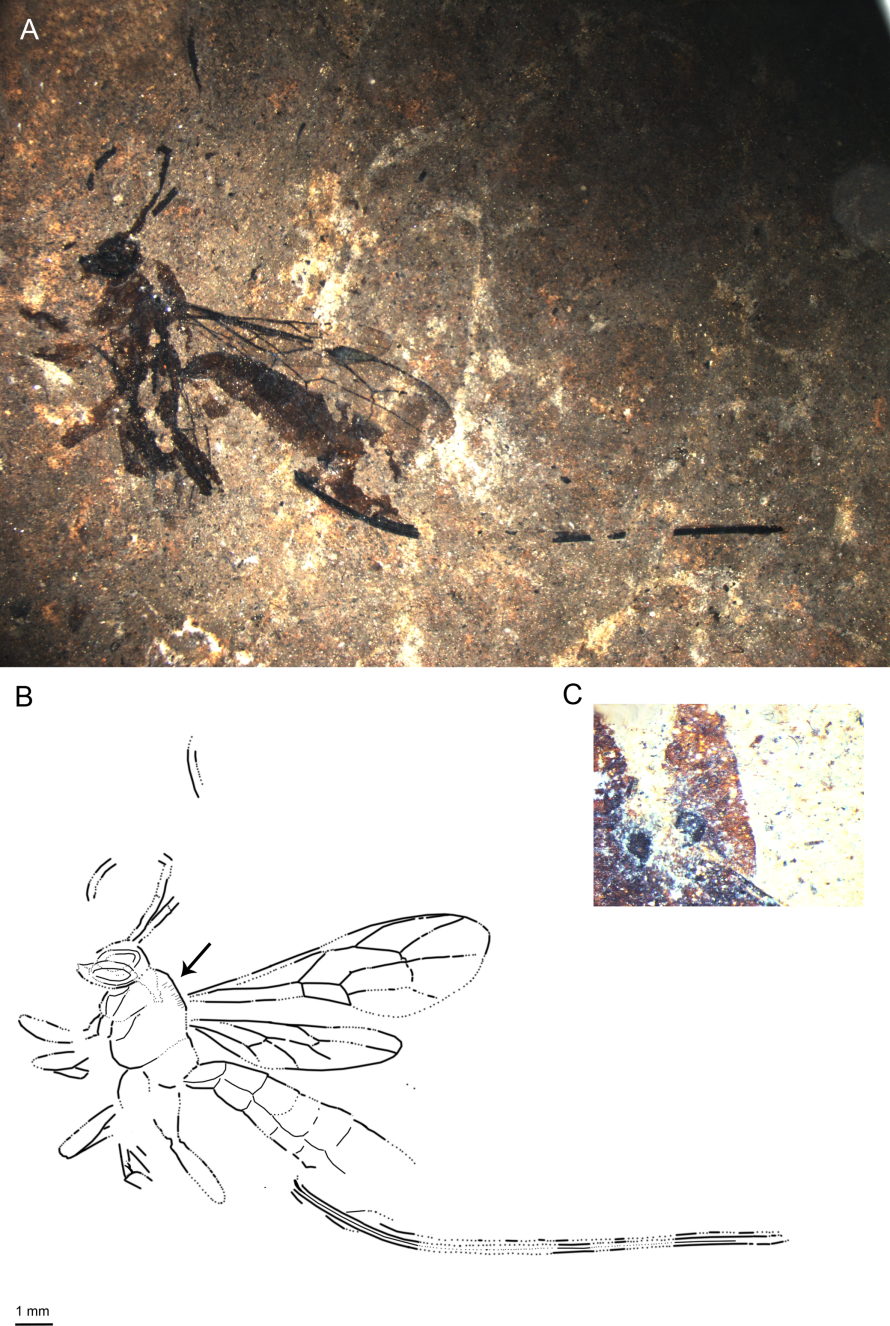
*Rhyssella vera* sp. nov., holotype SF MeI 8814. (A) Photograph. (B) Drawing; fore and hind wing were separated in the drawing; dashed lines indicate uncertain interpretations, black arrow indicates mesoscutum. (C) Details of the mesoscutum rugose sculpture.

#### Etymology

From Latin “vera” which means “real” or “true”.

#### Studied material

Holotype: SF MeI 8814, female. Lateral aspect of body with incomplete antennae, complete fore and hind wing, incomplete legs and incomplete ovipositor; parts of mesosoma and metasoma missing.

#### Type locality and horizon

Messel Pit Fossil Site (Hesse, Germany), Middle Messel Formation. The fossil originates from the former collection Behnke, details concerning digging site and horizon are unknown.

#### Systematic placement

Based on the rugose mesoscutum, long ovipositor and very short hind wing vein 1Cu, the specimen can firmly be placed in the subfamily Rhyssinae (as tribe Rhyssini in [2]). Considering the strongly petiolate areolet, the best fit is within the genus *Rhyssella* Rohwer or *Cyrtorhyssa* Baltazar. The fossil clearly differs from the genus *Cyrtorhyssa* in vein 2m-cu attached anterior to vein 3rs-m, 4Rs only slightly curved, shorter vein 2Cu in respect to 1M&1Rs and r-rs, and shorter T1 [2]. In contrast, the above mentioned characters support the placement of the fossil in the genus *Rhyssella*.

#### Diagnosis

Mesoscutum with transversal rugae. Ovipositor long, around 1.2x as long as forewing. Hind wing 1Cu almost absent or indistinct. Areolet very small, triangular,strongly petiolate. 2m-cu slightly bent in anterior half with two bullae. T1 short. Ground colour dark red-brown. Mandibulae stout, bi-dentate.

The fossil differs from most of the extant *Rhyssella* species in the less slender cell 2R1 and different shape of cell 2M, with vein 1m-cu shorter and not curved at the base (in the extant *R*. *humida*, 1m-cu is a bit shorter and more straight, approaching the state in *R*. *vera*).

#### Description

Ground colour dark red-brown. Antennae and wing veins dark brown to black. Legs of similar colour as most of body; fore and mid leg femur and tibia lighter. Ovipositor and its sheaths black.

Head with antennae incomplete, at least 12 flagellomeres visible; first flagellomere 3.2x as long as broad; scape distinctly longer than pedicel. One mandible present, stout and with two teeth of more or less same thickness; surface of mandible with strong punctures, separated by less than their diameter. Malar space around 0.4x base of mandible.

Mesoscutum with some transversal rugae visible especially in posterior half; indication of notauli might be present. Pronotum about as high as long. Epicnemical carina extending at least until mid-height of pronotum, not clear if curving anteriorly. Propodeum short, only partly preserved.

Forewing with areolet very small, triangular and strongly petiolate, 2Rs 1x 2 + 3M and 0.6x 3rs-m, 4M absent. 4Rs moderately sinusoidal. Pterostigma 4.4x as long as deep, 0.8x 1R1. 2m-cu slightly bent in the upper half with two bullae. 2M cell elongated; 1m-cu&2Rs + M angled; 1Rs + M absent or very short. 1cu-a slightly posterior from junction of M + Cu and 1M. 3Cu around as long as 2cu-a. Hind wing 1Cu almost absent or indistinct; M + Cu evenly bent.

Legs incompletely preserved. Mid leg with long tibial spurs, one slightly longer than other. Hind coxa somewhat elongate, femora rather slender.

Metasomal T1 1.2x T2, in lateral aspect with hump in first half; T2 subqadrate to slightly elongate; remaining tergites transverse (but most apical tergites missing). Ovipositor with incomplete tip, long and straight, at least around 1.2x as long as forewing; ovipositor sheaths with short dense hairs.

Measurements: forewing = 8.6 mm, width = 2.6 mm; body ≈11.7 mm; metasoma ≈7.9 mm; T1 = 1.2 mm; T2 = 1.2 mm; ovipositor >13.8 mm; ovipositor sheaths >11.1 mm.

Subfamily **Labeninae** Ashmead, 1900 [62]

Genus ***Trigonator*** gen. nov

urn:lsid:zoobank.org:act:FCF88625-2740-40C6-B3D4-662FC333B381

#### Etymology

From the Latin word for triangle “trigonus” that alludes to the triangular cell 2M in the forewing of the fossil and from the ending “-tor” that was suggested but not consistently applied as a species ending for all the ichneumonids; gender masculine.

#### Type species

***Trigonator macrocheirus*** sp. nov.

#### Systematic placement

The specimen is clearly placed in Labeninae due to the wide pentagonal 1+2Rs cell and high attachment position of the metasoma on the propodeum [63]. Within the subfamily, the fossil mostly resembles some genera in the tribe Labenini Ashmead and Poecilocryptini with the shape of T1 and mesosoma, proportions of 1+2Rs cell and slender hind legs. The coxal insertions in the same plane as the metasomal insertion would point to Labenini [63], but the interpretation of this character is somewhat uncertain and thus we refrain from a tribal placement. Vein 3Cu is never missing in any of the extant genera of the subfamily [2] (although this is frequent in the subfamily Ophioninae); we thus place the fossil in a newly described genus.

#### Diagnosis

Areolet pentagonal and around 2x as long as wide; vein 3Cu absent, 2M cell thus triangular. Metasoma attached to mesoscutum clearly higher than coxae; coxal insertions almost in same plane with metasoma attachment point. Ovipositor around as long as forewing or longer. Hind wing with vein 1Cu slightly longer than cu-a; 1Rs longer than rs-m.

***Trigonator macrocheirus*** sp. nov.

urn:lsid:zoobank.org:act:853AF27A-7C08-435C-8B71-CD23B160DC68

(Fig 6)

**Fig 6:**
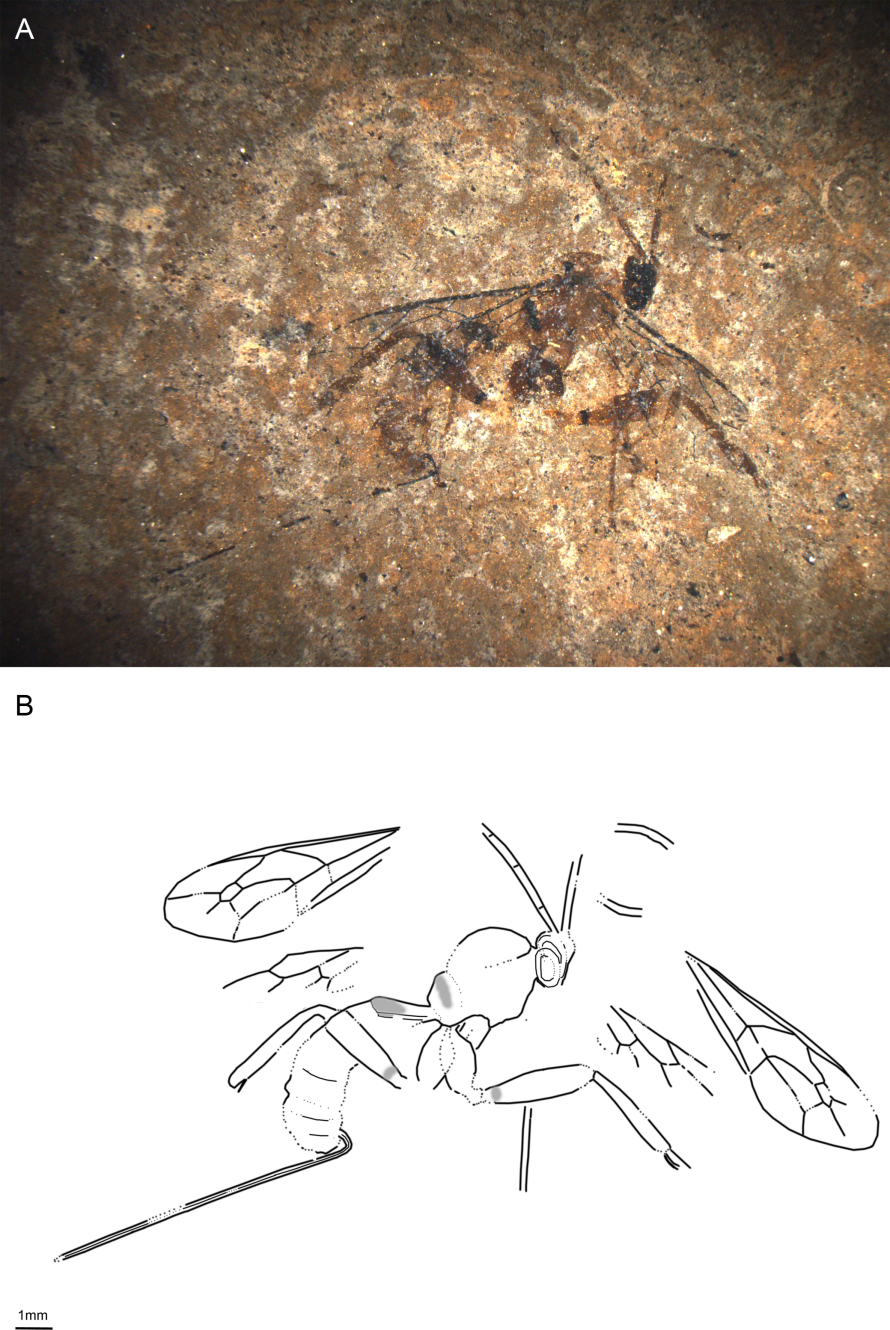
*Trigonator macrocheirus* gen. et sp. nov., holotype SF MeI 17304. (A) Photograph. (B) Drawing; left and right fore and hind wings were separated in the drawing; dashed lines indicate uncertain interpretations, while grey areas indicate colour patterns.

#### Etymology

Named after the Japanese spider crab genus *Macrocheira*; the fossil resembles these crabs with its very long and spread hind legs.

#### Studied material

Holotype: SF MeI 17304, female. Dorsolateral aspect of body with incomplete antennae, fore and hind wings, hind legs with parts of tarsi and parts of mid legs; mesosoma and metasoma preserved without any details.

#### Type locality and horizon

Messel Pit Fossil Site (Hesse, Germany), Middle Messel Formation. Details for holotype SF MeI 17304: grid square F 8/9, 2.05m to 2.5m above local stratigraphic marker level alpha.

#### Diagnosis

As for the genus by monotypy.

#### Description

Ground colour orange-brown. Antennae orange-brown, darker towards apex; head dark brown. Mesosoma with dark markings in front of scutellum, at base of forewing and at base of propodeum. Wing venation dark brown, vein C and pterostigma orange-brown. Hind legs dark orange-brown; trochanters orange-brown; hind femora narrowly dark at base and orange-brown at apex; hind tibia a bit darkened at apex. Metasoma orange-brown, apex of first, second, and maybe third tergite darkened. Ovipositor brown.

Head rather short and its back slanting steeply. Outline of eye discernible towards back, with gena very narrow. Antennae incomplete, at least 1.1x forewing; scape and pedicel of normal dimensions; basal segments elongate, first flagellomere 4.45x as long as wide, last visible segments wider than long.

Mesosoma poorly preserved, no clear limits between sclerites or carinae discernible. Mesoscutum short and rounded in profile. Indication of notauli might be present.

Forewing rather elongate, with venation complete. C and Sc + R adjacent, no cell in between. Areolet pentagonal and 2.2x as long as wide; 2Rs 0.5x 3Rs, 0.5x 2 + 3M and 1x 3rs-m, 2 + 3M 2.4x 4M; 2m-cu meeting areolet close to its outer corner. Vein 1cu-a meeting M + Cu very slightly anterior to junction of 1M and 2Cu. Vein r-rs sinuous, vein 1m-cu&2Rs + M evenly curved, no indication of 1Rs + M. Either one wide or two narrow bullae in the lower part of 2m-cu. Vein 3Cu absent, 4Cu meeting 2Cu directly, cell 2M thus triangular. Hind wing 1Cu/cu-a ratio 1.2; vein M + Cu straight; 1Rs slightly bowed, 1.6x rs-m.

Hind coxa large, around 1.6x as long as high. Hind femur around 3.6x as long as wide, without ventral tooth. Hind tibia slender, 6.4x as long as wide; with two slender spurs, inner spur bit shorter than outer spur. Shadow of what might be mid tibia and tarsomeres discernible.

Metasoma with first segment elongate, possibly petiolate. Attachment point to mesosoma clearly high above point where coxae meet propodeum; coxal insertions almost in same plane with attachment point. Ovipositor straight and slender, at least 1.2x as long as metasoma.

Measurements: antennae >8.6 mm; forewing = 7 mm, width = 2.3 mm; body = 10.1 mm; metasoma = 6.2 mm; T1 = 1.7 mm; ovipositor >8.2 mm.

Ichneumonidae *incertae subfamiliae*

Genus ***Polyhelictes*** gen. nov.

urn:lsid:zoobank.org:act:EECCD69A-E04F-464A-A2B1-590076D86694

#### Etymology

Named after the two genus-groups, *Polysphincta* and *Helictes*, to which the fossil might belong; gender masculin.

#### Type species

*Polyhelictes bipolarus* sp. nov.

#### Systematic placement

The fossil belongs either to the *Polysphincta* genus-group (tribe Ephialtini, subfamily Pimplinae) or to the *Helictes* genus-group (subfamily Orthocentrinae) according to its size, wing venation–especially obliterate vein 2Rs of the areolet, T1 shape, ovipositor length and dimensions of flagellomeres [2]. It could also belong to Cryptinae based on these characters, but in this subfamily, the base of T1 is always distinctly thinner than in the fossil and other two subfamilies. In favour of the *Polysphincta* genus-group are the shiny hind margins of T2–T4 (cf. *Acrodactyla* Haliday [51]) and the ovipositor thickness [2,9,10]. However, the more complete propodeal carination and rugose sculpture of T1 rather point to the *Helictes* genus-group. Since no clear distinction between the two is possible with the available characters, and no genus in either group shows a full match with this specimen, we describe a new genus within Ichneumonidae *incertae subfamiliae*.

#### Diagnosis

T2–T4 transverse, with hind margin shiny black. T1 broadly attached but still diverging posteriorly; strongly sculptured, with longitudinal rugae. Propodeum with several delimitated areas. Areolet open, 2Rs almost obscured, 2 + 3M rather long. Ovipositor straight, quite stout, sheaths about 0.24x as long as forewing. Hind wing vein M + Cu bowed between middle and end; 1Rs as long as or slightly longer than rs-m; vein cu-a very short or 2Cu absent. Mesoscutum with long notauli.

***Polyhelictes bipolarus*** sp. nov.

urn:lsid:zoobank.org:act:13541EF3-7F4D-4FA1-ABAB-D9A44EAF2DBD

(Fig 7)

**Fig 7:**
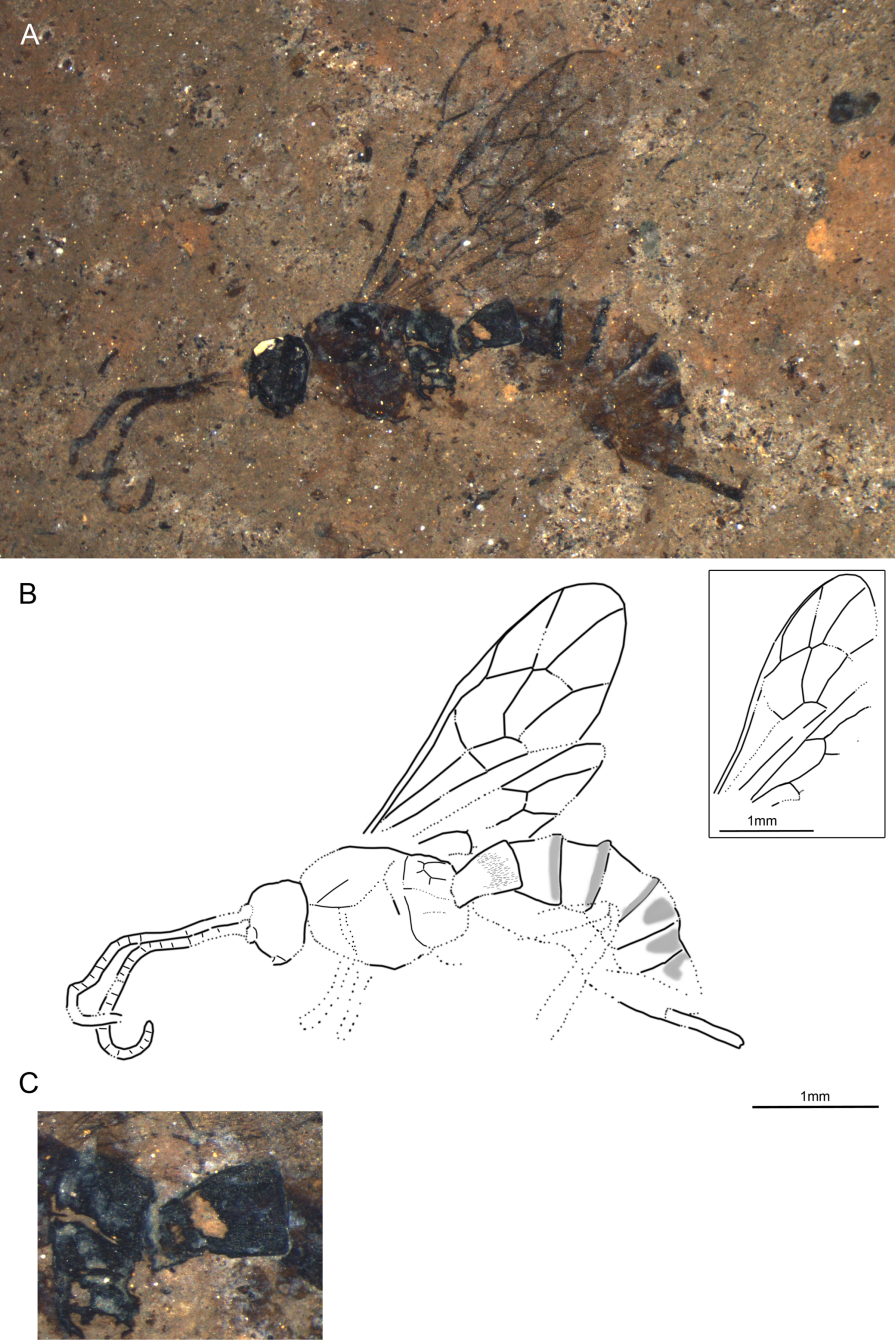
*Polyhelictes bipolarus* gen. et sp. nov., holotype MeI 16069. (A) Photograph. (B) Drawing; left and right fore and hind wings were separated in the drawing; dashed black lines indicate uncertain interpretations, grey lines structure on tergite 1, and grey areas colour pattern. (C) Details of propodeum carination and tergite 1 sculpture.

#### Etymology

From word “bipolar” which additionally emphasises duality of the taxonomic position of the fossil.

#### Studied material

Holotype: MeI 16069, part, female. Lateral aspect of body with almost complete antennae, fore and hind wings, traces of hind and front leg, ovipositor sheaths and partial ovipositor.

#### Type locality and horizon

Messel Pit Fossil Site (Hesse, Germany), Messel Formation. Details for holotype MeI 16069: grid square F9, 2.5m to 3.5m above local stratigraphic marker level alpha.

#### Diagnosis

As for the genus by monotypy.

#### Description

Head black, antennae dark brown. Pronotum, propleuron, mesoscutum and mesopleuron orange-brown; propodeum black. Wing veins dark brown, pterostigma lighter medially. Legs orange or yellow. T1 black; T2–T4 orange with narrow, black end margins, T2 additionally with irregular brown markings; T5–T7 orange with black mark medially. Ovipositor sheaths brown.

Head roundish, no details discernible. Antennae almost complete, with about 20 flagellomeres; most flagellomeres rather stout, median ones about 1.3x as long as wide; scape and pedicel unclear.

Mesoscutum with long notauli. Pronotum moderately long with epicnemical carina extending at least to mid-height of pronotum. Sternaulus-area unclear. Propodeum with pleural carina, split where lateral longitudinal carina might be, and traces of probably middle part of basal transverse carina; broken off where posterior transverse carina might be; certainly with several delimited areas; submetapleural carina complete.

Forewing vein rs-m absent (areolet open), 2Rs almost obscured, 2 + 3M rather long. Pterostigma around 4.1x as long as wide, 0.8x 1R1. 2Cu 0.8x 1M + 1Rs, 0.8x r-rs. 1m-cu&2Rs + M angled or curved. 1cu-a at junction of M + Cu and 1M. 3Cu 1.2x cu-a. 2m-cu slightly and evenly curved with one or two bullae. Hind wing 1Rs around 1.1x rs-m; vein cu-a very short or 2Cu absent; vein M + Cu bowed between middle and end.

Fore and hind leg weakly indicated, hind leg seems rather slender, maybe with two spurs on hind tibia.

Metasoma probably slightly compressed. T1 broadly attached but still diverging posteriorly, 1.5x T2; strongly sculptured, with longitudinal rugae. T2–T4 wider than long, with hind margin shiny black. T5–T7 rather shorter. Last sternites visible, hypopygium short and straight. Ovipositor straight, quite stout, sheaths about 0.24x as long as forewing.

Measurements: antennae = 2.2 mm; forewing 2.9 mm, depth = 1 mm; body = 4.2 mm; metasoma = 2.5 mm; T1 = 0.6 mm; T2 = 0.4 mm; ovipositor sheaths = 0.7 mm.

Ichneumonidae *incertae subfamiliae*

Genus ***Mesornatus*** gen. nov.

urn:lsid:zoobank.org:act:3291094F-73AB-49F8-9F0D-ED030DC89EAA

#### Etymology

From prefix “meso-” which refers to the mesosoma and the Latin word “ornatus” which means “distinguished”; gender masculine.

#### Type species

***Mesornatus markovici*** sp. nov.

#### Systematic placement

This genus cannot be placed in a single ichneumonid subfamily. Considering the size, wing venation (especially the areolet with a very short vein 2 + 3M and long vein 2Rs, and the high 1Rs/rs-m ratio in the hind wing), and the shape of metasoma and T1, the fossil could belong to Ctenopelmatinae, Banchinae or Tryphoninae [2,46]. However, most Tryphoninae and Banchinae do not have a transverse T2, and Banchinae only rarely have such long carinae on T1. On the other hand, Ctenopelmatinae often have vein M + Cu bent in the hind wing [2]. Since there is no extant genus in any of the three subfamilies that would match our fossil, also considering the very distinct colour pattern on the mesosoma, we define a new genus with uncertain subfamily placement.

#### Diagnosis

Mesoscutum with distinct colour pattern—ground colour black, orange-brown along extension of notauli on posterior half, sides of scutellum, postscutellum and possibly end of propodeum; very heavily punctured with very deep notauli meeting at mid-length. Prescutellar groove with strong carinulae; axillar troughs of mesonotum and metanotum well-delimited, the former with transverse carinulae. Areolet open; 2M cell sub-quadrate, vein 1Rs + M present. Hind wing 1Cu/cu-a ratio around 2.8. T1 short and stout, evenly tapers towards front; with strong and evenly converging dorsal longitudinal carinae and strong and dense punctures on most of surface and diagonal rugae on sides of longitudinal carinae on basal half. T2 and following tergites transverse and heavily punctured. Metasoma fusiform.

***Mesornatus markovici*** sp. nov

urn:lsid:zoobank.org:act:E932E7C4-932E-4CDB-A3D4-9251BABDCD52

(Fig 8)

**Fig 8:**
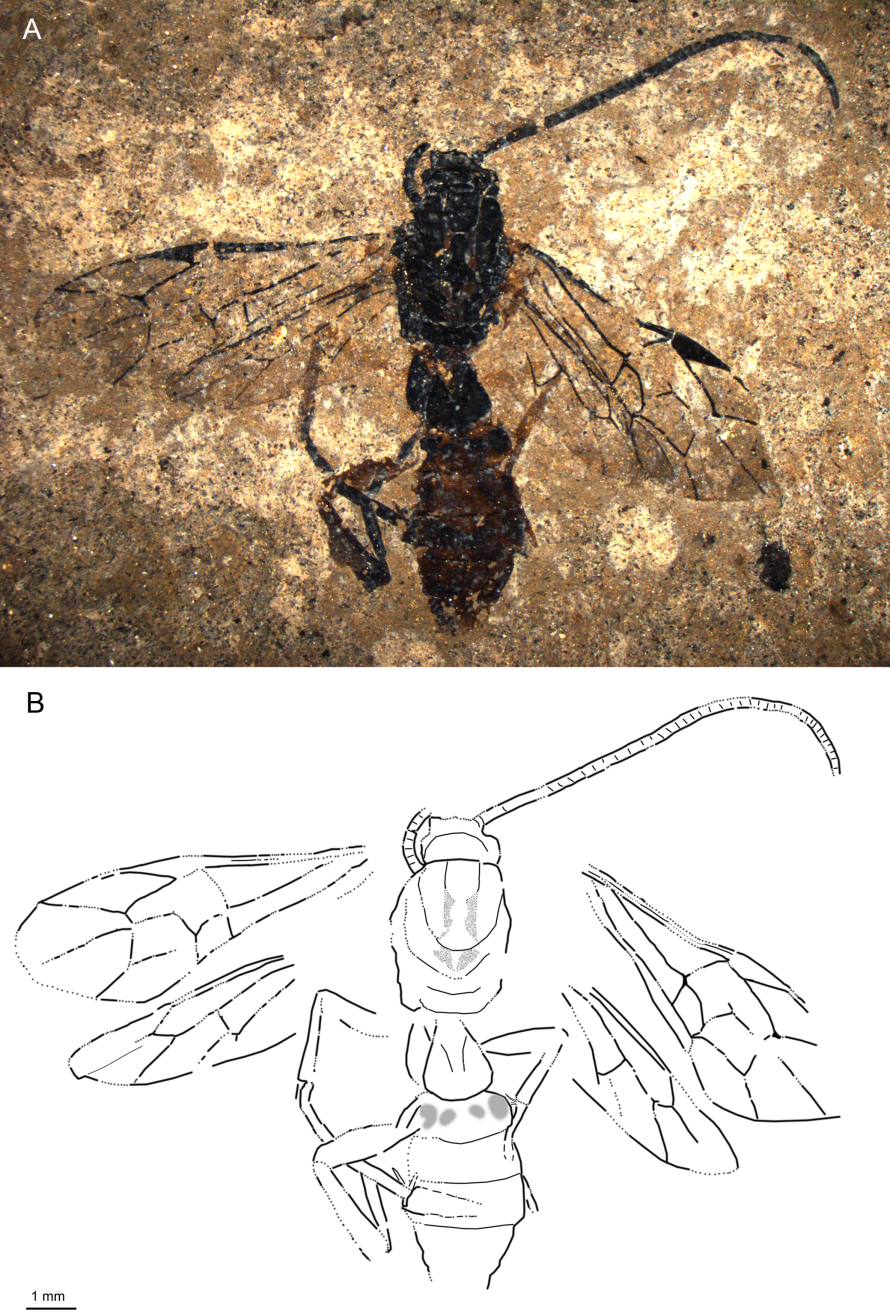
*Mesornatus markovici* gen. et sp. nov., holotype SF MeI 15245. (A) Photograph. (B) Drawing; left and right fore and hind wings were separated in the drawing; dashed lines indicate uncertain interpretations, while grey areas indicate colour patterns.

#### Etymology

Dedicated to a dear friend and one of the most enthusiastic and gifted biologists I (TS) have ever met. Like the fossil, Djordje Markovic is a remarkable specimen, seeking–and hopefully one day finding–his position in the scientific system.

#### Studied material

Holotype: SF MeI 15245, female (?). Dorsal aspect of body with fore and hind wings, one complete antenna, parts of mid and hind legs; without ovipositor.

#### Type locality and horizon

Messel Pit Fossil Site (Hesse, Germany), Middle Messel Formation. Details for holotype SF MeI 15245: grid square F 9, 3.5m to 4.5m above local stratigraphic marker level alpha.

#### Diagnosis

As for the genus by monotypy.

#### Description

Ground colour black. Orange along extension of notauli on posterior half of mesoscutum, sides of scutellum, postscutellum and possibly end of propodeum. Hind coxa, apex of tibia and tarsi black, hind tibia dark brown; hind femur, mid and front legs orange-brown.T1 black, remaining tergites orange-brown, T2 with four black lateral markings.

Head with vertex distinctly punctured, gena short; occipital carina strong and evenly convex. Antennae 1.13x forewing length, with at least 47 flagellomeres; segments very short, apical ones twice as wide as long, first flagellomere 2.5x as long as wide; fine hairs visible. Shape of scape and pedicel indistinct.

Mesoscutum very heavily punctured on smooth and polished background; notauli very deep, parallel on anterior half of mesoscutum then either strongly converging around middle or extending parallel until end of mesoscutum; carinae along lateral margin uninterrupted from tegula to scutellum. Prescutellar groove with strong carinulae; Axillar troughs of mesonotum and metanotum well-delimited, the former with transverse carinulae. Propodeum quite short, heavily punctured, with at least basal portion of median longitudinal carinae and anterior transverse carina present; pleural carina probably present as well.

Wing veins somewhat distorted. Forewing with areolet open, vein 2 + 3M absent or as short as width of surrounding veins. Pterostigma around 3.4x as long as wide, 0.69x 1R1. 2Cu 1x 1M&1Rs, 0.7x r-rs. Veins 1m-cu and 2Rs + M meeting at an angle where 1Rs + M (ramulus) originates; 1Rs + M around 4x as long as width of vein 1m-cu. 1cu-a meeting 2Cu posterior of the junction M + Cu and 1M. 2cu-a seems almost absent, but this is likely an artefact (vein 1m-cu broken low and 3Cu and 2cu-a are moved downwards). Hind wing 1Cu/cu-a ratio 2.8x; vein 2Cu weak; M + Cu mostly straight, slightly curved proximally; cell 1Cu rather long.

Hind femur about 3.7x longer than wide. Hind tibia robust, becoming thicker towards apex, 4.3x as long as wide, very hairy and with fringe of strong, short bristles and two long spurs at apex. Hind tarsomere very elongate and robust and hairy.

Metasoma quite fusiform. T1 about 1.2x as long as apically wide, evenly tapering towards front; with very strong and evenly converging dorsal longitudinal carinae; with very strong and dense punctures on most of surface and diagonal rugae on sides of longitudinal carinae on basal half, area between carinae possibly smooth in basal half. T2 and following tergites more than 2x wider than long, heavily punctured. No ovipositor visible; strong fusiform shape of metasoma suggests female.

Measurements: antennae = 8.9 mm; forewing = 7.7 mm, width = 2.8 mm; body = 9.9 mm; metasoma = 5.7 mm.

## Discussion

The Messel Pit is known as a locality with unique fossil preservation and many remarkable fossil findings [18]. Here we described the first ichneumonids from the locality, which unsurprisingly show good preservation that in most cases allows their confident placement in recent subfamilies and genera.

### First fossilized lobed claws

By far the largest number of described Eocene ichneumonid fossils have been placed in the subfamily Pimplinae [6], but the taxonomic position of many of them is actually uncertain and often based on superficial resemblance to extant representatives of the group [7,64–66]. The uncertainty partially comes from the plesiomorphic nature of many pimpline body and wing venation character states, such as the stout tergite 1 with dorsal longitudinal carinae and the quadrate areolet [51,67]. Even the subfamily placement of extant pimpline males can be difficult without having corresponding females [52,68]. Moreover, the suggested synapomorphies for the subfamily, e.g., the thyridium on T2 sunken in a deep transversal groove, hind margin of the tergite of females sculpturally different than rest of tergite, and a medially divided tergite VIII/XI in males [51], are usually not preserved in fossils.

We therefore consider the here described *Scambus fossilobus* as the first unequivocal record of the subfamily Pimplinae from the Eocene. The placement of our fossil in the subfamily is evidenced by the presence of lobed claws, a character state that supports the monophyly of at least a subset of genera in the subfamily Pimplinae [51] and has never been documented before in a fossil. Although lobed claws are occurring in some Labeninae (tribe Poecilocryptini), Orthopelmatinae and Collyrinae [46,51], the combination of characters observed in the specimens from Messel points clearly to the subfamily Pimplinae (see “Systematic placement” section for *Scambus fossilobus*)

### The oldest Rhyssinae

*Rhyssella vera* represents the oldest unambiguous fossil of the subfamily Rhyssinae. Three other rhyssine fossils have been described previously: *Rhyssa antiqua* Heer [69] from the Early Miocene locality Radoboj in Croatia, *R*. *petiolata* Brues [70] from the Late Eocene [71] Florissant Formation, and *R*. *juvenis* Scudder [66] from the Early Eocene of Fish-Cut, Greater Green River Basin, Green River Formation [72], both in the US. *R*. *antiqua* has been placed in the subfamily based on the forewing length (~18mm) and wing venation patterns that resemble the ones found in extant *R*. *persuasoria* Linnaeus (see page 36 in Heer [69]). However, there are a couple of genera in the related subfamilies Pimplinae and Poemeninae that can have very similar wings. Moreover, the wing venation pattern in *R*. *antiqua* is inconsistent between the two wings in the drawing given in the original description, especially with respect to the shape of the areolet, which appears too broad for Rhyssinae, and the ratio of hind wing veins 1Cu and cu-a, which is too large for the subfamily (see Taf. III, Fig 18 in Heer [69]). In the case of *R*. *juvenis*, the absence of clear characters for subfamily placement has already been discussed in Spasojevic et al. [7]. *R*. *petiolata* is placed in Rhyssinae based on more reliable characters, such as rugae on the mesoscutum and a long ovipositor (9mm) [70]. However, Brues [70] also reported an areolated propodeum in the fossil, which does not occur in extant Rhyssinae. In any case, this fossil is from the latest Eocene and thus considerably younger than the here described *R*. *vera*.

### Biogeographic history of Labeninae

**Fig 9:**
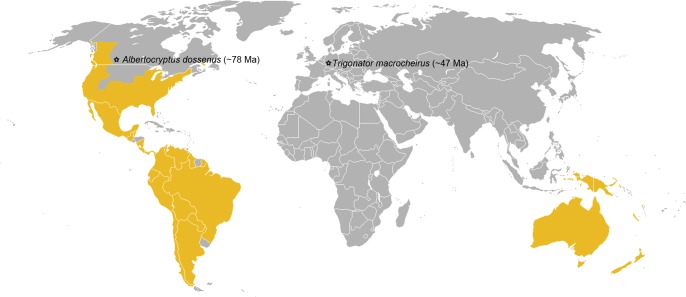
Distribution map of the ichneumonid subfamily Labeninae. Labenine today show a primarily Gondwanan distribution, but they are absent from African, India and Madagascar and present in the southern parts of North America. Stars indicate localities of the labenine fossil record, which suggest a more global distribution of the subfamily in the past; the approximate ages of the fossils are given in brackets. Image from the public domain (https://commons.wikimedia.org/wiki/File:BlankMap-World.png) modified based on distribution record from Yu et al. [1] and reprinted under a CC BY licence.

The newly described *Trigonator macrocheirus* is the first certain labenine fossil and the only one from Eurasia (Fig 9). The only previously described fossil of this subfamily, *Albertocryptus dossenus*, shows some untypical characters when compared to recent representatives of the subfamily, such as small size, an elongate scape and short antenna with only 19 flagellomeres, a very wide and short pterostigma, and a strongly humped T1 in the shape of a petiole (even though a similar state occurs in the genus *Labium* in the tribe Groteini). Therefore, even though they placed the fossil in the recent tribe Poecilocryptini, McKellar et al. considered erecting a new tribe within Labeninae or even a new subfamily for it [13]. In contrast, *T*. *macrocheirus* has the typical appearance of recent labenine genera, which indicates its position within the crown group of Labeninae.

The presence of a crown group Labeninae in Eurasia during the early Eocene contradicts the notion that this subfamily radiated on western Gondwanaland approximately 80 million years ago (Late Cretaceous) as it was suggested by Gauld & Wahl [63] based on present-day distribution patterns. But it supports, together with the McKellar et al.’s [13] labenine record in the Canadian amber (~78 Ma), a more global distribution of Labeninae during the Late Cretaceous, while the current predominantly Gondwanan distribution would then simply represent a relict distribution. This complies with not rare fossil findings of “Gondwanan” insect taxa in Eurasia and North America [73,74].

The global distribution of Labeninae during the Late Cretaceous further implies diversification and spread of the group already during Early Jurassic/Late Triassic (>200 Mya), before the separation of Europe from Laurentia-Gondwana. However, the oldest clear ichneumonid fossils from the extinct subfamily Palaeoichneumoninae are only between 137 and 121 Ma old (Early Cretaceous) [4,8], and up until now, there is no fossil evidence that any of the extant subfamilies was already present during that period. The available age estimates for the family Ichneumonidae, based on phylogenetic methods, are around 140 Ma [75] and 190 Ma [76], but they stem from dating studies where ichneumonids were not a focal group and are thus rather unprecise due to low taxon sampling. Therefore, a dated phylogeny of the family with age estimates for the subfamilies is essential to resolve the timing of the labenine radiation.

Alternatively, the diversification of Labeninae occurred during the Early Cretaceous and the group dispersed either via existing land bridges [77–79], as suggested for many insect groups [80–84], or by a long-distance dispersal. Ichneumonid wasps are today and have probably always been very mobile organisms [85–88] with comparatively low levels of endemism [1,2,89,90]. The dispersal abilities of Labeninae thus might be high enough that trans-oceanic dispersal is a real option, which would already have erased any signal of past distributional patterns, especially those as old as Cretaceous vicariance events. Nevertheless, a more complete fossil record of labenine is needed to infer the dispersal pathways.

#### Labeninae and Labenopimplinae

A link could possibly be made between Labeninae and the extinct, Late Cretaceous subfamily Labenopimplinae. Labenopimpline are a morphologically heterogeneous assemblage of ichneumonid fossil genera with mixed characteristics also found in modern Labeninae and Pimplinae [12,91,92]. They have been described from Russia [12,92], Canada [13] and Botswana [91], which reflects their wide distribution range during the Late Cretaceous (see checklist in Li et al. [11]). If the subfamily were indeed an ancestral lineage to Labeninae as suggested by Kopylov [12], the hypothesis of an initial wide distribution of Labeninae linked to high dispersal abilities would be supported. However, the two subfamilies clearly differ in the shape of T1 and the attachment point of the metasoma, and their relationships remain rather unclear; thus any conclusion would be speculative.

### (Sub)tropical genus from the European Eocene

**Fig 10:**
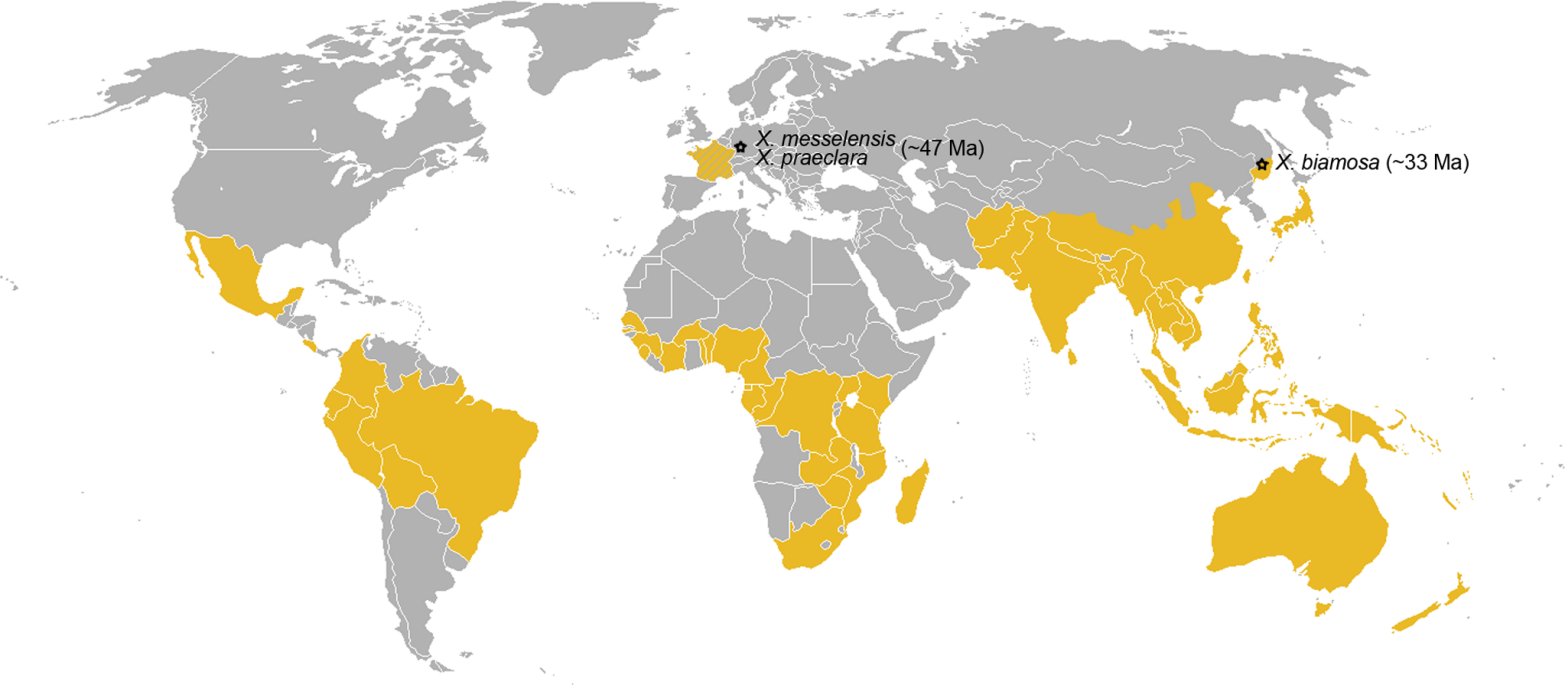
Distribution of the subtropical genus *Xanthopimpla*. *Xanthopimpla* is nowadays widely distributed in tropical and subtropical areas of the world. Striped area indicates uncertain occurrence of the genus in France, while stars indicate localities of the fossil record; approximate age of the fossils is given in brackets. Image from the public domain (https://commons.wikimedia.org/wiki/File:BlankMap-World.png) modified based on distribution record from Yu et al. [1] and reprinted under a CC BY licence.

*Xanthopimpla* is the largest genus of the subfamily Pimplinae, with a wide distribution in the tropics and subtropics (Fig 10) [2,57,58]. The species richness is highest in the Indo-Australian and Oriental regions, while a few species can be found in Africa and in the Neotropics [57,58]. Because of its current distribution, the genus is considered to indicate a warm-temperate and subtropical climate [59]. The two newly described *Xanthopimpla* species support this observation. In the Eocene, the climate around former Lake Messel was warm and humid. The mean annual temperature was about 22°C, the mean temperature of the coldest month was above 10°C and the mean annual precipitation reached up to 2540 mm [93]. The vegetation around the former Lake Messel seems to be have been a multilevel canopy forest with a large tropical-paratropical component, and large part of the fauna consisted of subtropical and tropical elements [25,34].

Additionally, the fossils provide evidence that the genus is at least of Early/Middle Eocene age, while the only previously known fossil of *Xanthopimpla* is *X*. *biamosa* Khalaim [59] from the Late Eocene locality Biamo in Russia [94].

### The unplaceables and taxonomic uncertainty

The taxonomic placement of incompletely preserved fossils can be very challenging due to a lack or uncertain interpretation of visible synapomorphies [10]. However, fossils from the Messel Pit often show remarkable preservation with many morphological details retained [11,12]. Although this was the case in the newly described ichneumonid fossils, we were not able to firmly place two of them. Both *Polyhelictes bipolarus* and *Mesornatus markovici* show character state combinations that occur in more than one extant ichneumonid subfamily. This issue has already been reported for ichneumonid fossils and is mostly attributed to the high rates of homoplasy observed in these parasitoids [7,13]. A phylogenetic analysis with combined morphological data from extant and fossil ichneumonids could possibly resolve taxonomic position of such taxa—especially of *P*. *bipolarus* where the analysis of a full morphological character set may confidently place the fossil in one of the two potential subfamilies or even as a stem lineage of one of the two.

## Conclusions

The unique fossil preservation in the Eocene Messel Pit allows us to firmly place several ichneumonid fossils in extant subfamilies and genera. As a result, we report the oldest unequivocal representatives of the subfamilies Pimplinae, Rhyssinae and Labeninae and of the genus *Xanthopimpla* (which was up till now only known from the latest Eocene). Although the new findings significantly increase our knowledge on the age of ichneumonid parasitoids, they also raised new questions, especially regarding the radiation of Labeninae. The previous hypothesis of a Gondwanan radiation after the initial break-up of the supercontinent is disproved by the newly described *Trigonator macrocheirus*, the first labenine record from Europe and the second from a non-Gondwanan continent. The observed pattern in their fossil distribution could be explained either by an older radiation of the group or by their high dispersal ability, which allowed them to cross existing water barriers during the Late Cretaceous and/or the Paleogene. However, this complex biogeographical question requires a better knowledge of the fossil record and rigorous age estimation for the subfamily. A solid, dated phylogeny of Ichneumonidae, resulting from an integrative analysis of molecular and combined extant and fossil morphological data, would not only provide much-needed age estimates for the subfamilies, but would also help resolving some uncertain taxonomic positions and facilitate placement of ichneumonid fossils in the future.
